# Evaluation of clinical usability and reproducibility of the American Society for Reproductive Medicine Müllerian Anomalies Classification

**DOI:** 10.1016/j.eurox.2025.100425

**Published:** 2025-08-26

**Authors:** Julia Lastinger, Natalia Palasz, Peter Oppelt, Galymzhan Toktarbekov, Milan Terzic, Raimund Stein, Katharina Rall, Helga Wagner, Philipp Hermann, Stephanie Kiblboeck

**Affiliations:** a Department of Gynecology, Obstetrics and Gynecological Endocrinology, Kepler University Hospital, Johannes Kepler University, Altenbergerstrasse 69, Linz 4020, Austria; b Clinical Academic Department of Women’s Health, Corporate Fund “University Medical Center”, Kerey and Zhanibek Khandar Street 5/1, Astana 010000, Kazakhstan; c Department of Surgery, School of Medicine, Nazarbayev University, Kerey and Zhanibek Khandar Street 5/1, Astana 010000, Kazakhstan; d Department of Pediatric, Adolescent, and Reconstructive Urology, Mannheim Medical Faculty, Mannheim University Medical Center, Heidelberg University, Theodor-Kutzer-Ufer 1-3, Mannheim 68167, Germany; e Department of Gynecology and Obstetrics, Tübingen University Hospital, Calwerstraße 7, Tübingen 72076, Germany; f Department of Applied Statistics, Johannes Kepler University, Altenbergerstrasse 69, Linz 4020, Austria; g Center for Clinical Studies (CCS Linz), Johannes Kepler University, Altenbergerstrasse 69, Linz 4020, Austria

**Keywords:** Female genital malformation, Müllerian anomaly, Uterine malformation, Classification system, ASRM MAC2021, Gynecology

## Abstract

**Objective:**

To study the usability, reproducibility and practicality of the revised American Society for Reproductive Medicine Müllerian Anomalies Classification (ASRM MAC2021) compared to the formerly used American Fertility Society (AFS) classification. The main focus of this paper is to present clinicians’ assessment when using these classification systems and to explore possible benefits and disadvantages of the transformation of the AFS into the more recently published ASRM classification. To incorporate all commonly used classification systems, all cases were additionally classified using the ESHRE/ESGE and the VCUAM classification.

**Methods:**

An observational study using a questionnaire and opinion survey was conducted with the goal of comparing clinicians’ subjective usability and reproducibility of the AFS classification with the newly presented ASRM MAC2021 classification. Cases of female genital malformations encompassed a wide range of various anomalies, including vaginal, cervical and adnexal conditions and associated malformations.

**Results:**

The study investigators rated the ASRM MAC2021 classification better in relation to usability and reproducibility compared to the AFS classification. It significantly improved diagnostic precision for vaginal and cervical anomalies and expanded recognition of adnexal malformations. However, challenges remain in addressing associated malformations. Clinicians ranked the ASRM MAC2021 higher than the AFS classification. However, investigators rated the ESHRE/ESGE and VCUAM classifications better in the overall assessment of female genital malformations.

**Conclusion:**

The ASRM MAC2021 classification offers substantial improvements in diagnosing and managing female genital malformations, addressing limitations of the AFS system. With better usability and subjective reproducibility, further refinements are needed to fully address complex associated anomalies — emphasizing the importance of evolving classification systems.

## Highlights

• ASRM MAC2021 rated more practical and reproducible than AFS classification.
• Improved diagnosis of vaginal, cervical and adnexal malformations.
• Clinicians preferred ESHRE/ESGE and VCUAM over ASRM overall.

## Introduction

Female genital malformations are a diverse group of congenital conditions, affecting over 6 % of the female population worldwide. They are found more commonly in patients suffering from infertility or recurrent pregnancy loss [1], [2], [3], [4], [5]. These anomalies often require specialist expertise, posing diagnostic challenges for general providers. Clinical manifestations of müllerian anomalies vary and many patients remain undiagnosed for years [9], [10], [11], [12]. These cases often challenge health-care providers who are not specialists in the care of this patient group [9], [10], [13], [14]. Advances in gynecologic imaging have led to a better understanding of the various manifestations of female genital malformations [2], [6], [7], [8].

Various classification systems have been proposed after the first one was developed in 1979 [15]. The American Fertility Society (AFS) presented a classification in 1988 limited to the most common uterine anomalies [1], [15]. The authors at the time noted that “modification (was) likely in the future” [1].

Further classification systems include the VCUAM (Vagina Cervix Uterus Adnexa-Associated Malformation) classification, with greater flexibility in assessing each of the various affected compartments and included associated malformations, such as renal, neurological, cardiac or skeletal manifestations [16], [17]. The European Society of Human Reproduction and Embryology (ESHRE)/European Society for Gynaecological Endoscopy (ESGE) published another classification based on the uterine compartment [4]. Other classification systems, such as that of Acién et al., are determined by the embryological development of female genital tract malformations. An international consensus has not yet been reached on which classification should be used [13], [18].

The latest classification is the American Society for Reproductive Medicine (ASRM) Müllerian Anomalies Classification (MAC2021) published in 2021 based on a modification of the AFS classification, with the goal of improving the diagnosis and strengthening interdisciplinary communication [9]. The AFS system had originally introduced been as a classification based on malformations of the uterus [1]. Its limited number of subgroups did not allow the differentiation of vaginal, adnexal, or associated malformations. When the ASRM MAC2021 classification was implemented, the goal was to use the strengths of the already existing system and to simplify communication. Specifications on the measurement of malformations were added. By adding descriptive terms and graphic illustrations to the six categories of the AFS classification, nine main subgroups of female genital malformations were formed [9], [10], [19].

Some complex malformations are still unclassifiable in terms of the ASRM MAC2021 and some authors have argued that classifications are proposed without peer review. No external validation had been used before publishing the ASRM MAC2021 classification [20], [21].

To date, no studies have been performed assessing the clinical usability and reproducibility of the ASRM MAC2021 classification. Here we present a subgroup analysis from the prospective exploratory EVA Study (*E*SHRE/ESGE/*V*CUAM/*A*FS) [22]. The objective of this data analysis was to evaluate and compare expert clinician’s opinions on usability, practicality and reproducibility of the AFS and ASRM classification systems for müllerian anomalies in a multicenter setting, with secondary observations related to the ESHRE/ESGE and VCUAM systems.

## Materials and methods

Between December 2019 and February 2022, 65 women with genital malformations were enrolled in the EVA Study in six tertiary care centers internationally. The study was approved by each of the study centers’ local ethics committees and registered at the German Clinical Trials Register (DRKS00025001). All patients with female genital malformations who consulted one of the study centers were asked to participate in the study. Study recruitment for this analysis was stopped after the new ASRM MAC2021 classification was published. Written informed consent was obtained from all patients or their legal guardians. This project was conducted in accordance with the Declaration of Helsinki.

A questionnaire (supplementary material) and opinion survey were completed by one study investigator for each case. All study investigators were medical doctors working in departments specializing in diagnosis and treatment of patients with müllerian anomalies. All participating centers had a dedicated outpatient clinic specializing in the diagnosis and management of congenital malformations, ensuring that the involved clinicians routinely encountered and managed patients with female genital malformations as part of their clinical practice. Prior to participation, each study investigator was provided with the complete EVA Study description and a completion guide for the study documents. The questionnaires provided free text boxes for the investigators to add additional comments on missing features or suggestions for improvement after each classification. Each patient was classified using three classification systems: the ESHRE/ESGE, VCUAM, and AFS. After the AFS classification was revised by Pfeifer et al. [9], all patients enrolled before November 2021 were reclassified by the same investigators. In total, 51 patients in four study centers were reclassified using the ASRM classification. Two of the initial study centers of the EVA Study were not available for providing reclassification, which explains how the study population came about. In this study, *usability* of the classification refers to the extent to which healthcare professionals can readily understand, apply, and interpret the system in clinical settings, ensuring clear categorization and practical utility for diagnosis, communication and treatment planning in cases of female genital malformations. To assess usability, compartments of each classification system were rated by the study investigators using a five-point Likert-type rating scale, ranging from (1) very good to 5 (very bad) in order to answer the question “How could the malformation(s) be described?”. Investigators only rated usability for compartments where a malformation was present. In cases with an isolated uterine malformation, for example, usability of each classification system for other compartments was not evaluated. Additionally, the investigators were asked to rank the classifications according to clinical *practicality* by answering the question “Which classification is the most clinically practical? Assign the numbers 1–3.”, with number one being the most clinically practical classification and number three being the least clinically practical classification system. Finally, clinician’s opinion on *reproducibility* was rated similarly with the question “Do you think that another person could accurately reproduce the diagnosed deformity using the created classification item?”. The questionnaire was provided with a separate completion guide for the individual questions and can be found in the supplementary material.

### Statistical analysis

For the grading of malformations, absolute and relative frequencies were computed on the basis of the total number of malformations observed per classification type. Associations between nominal or ordinal variables and study centers were tested using Fisher’s exact test, and the Kruskal–Wallis test was employed to test for location differences among metric variables across centers. Stacked bar charts illustrate the overall grading and classification of malformations per classification system. Statistical analysis was carried out by the Competence Center for Clinical Studies Linz (KKS Linz) at Johannes Kepler University, Linz, using the R statistical software package (2021).

## Results

A total of 51 patients with female genital malformations were evaluated across multiple centers using clinical examination and ultrasound. Malformations were classified according to four established classification systems (ESHRE/ESGE, VCUAM, AFS and ASRM MAC2021). All of the study participants received a clinical interview and examination by one of the 10 investigators. Among the patients, eleven patients (21.6 %) underwent additional magnetic resonance imaging (MRI). Table 1 provides an overview of patient characteristics relative to the study centers.

**Table 1:** Baseline characteristics of patients and physicians.

	**Total (n = 51)**	**Center 1 (n = 32)**	**Center 2 (n = 4)**	**Center 5 (n = 11)**	**Center 6 (n = 4)**	**p-value**
**Age (Median, IQR) (Years)**	23.32 (11)	22.99 (9.9)	10.17 (21.5)	26 (13.5)	30.5 (4.8)	0.072
**Time to Diagnosis (Median, IQR) (Months)**	12 (40)	24 (54.5)	0 (0.75)	NA (NA)	9.5 (8.3)	0.043
						
**Specialty** of rating physician - Gynecology (n, %)	47 (92.2 %)	32 (100 %)	0 (0 %)	11 (100 %)	4 (100 %)	< 0.001
- Pediatrics (n, %)	4 (7.8 %)	0 (0 %)	4 (100 %)	0 (0 %)	0 (0 %)	
Number of investigators per center (n, %)	10 (100 %)	6 (60 %)	1 (10 %)	1 (10 %)	2 (20 %)	0.078
Number of patients rated per investigator (Median, IQR)	3 (8)	1 (10)	1 (1)	1 (1)	3 (2)	< 0.001
**Specialist**						< 0.001
- No (n, %)	20 (39.2 %)	20 (62.5 %)	0 (0 %)	0 (0 %)	0 (0 %)	
- Yes (n, %)	31 (60.8 %)	12 (37.5 %)	4 (100 %)	11 (100 %)	4 (100 %)	
**Gravidity**						0.820
- Gravidity = 0 (n, %)	39 (76.5 %)	25 (78.1 %)	4 (100 %)	7 (63.6 %)	3 (75 %)	
- Gravidity = 1 (n, %)	5 (9.8 %)	3 (9.4 %)	0 (0 %)	2 (18.2 %)	0 (0 %)	
- Gravidity ≥ 2 (n, %)	7 (13.7 %)	4 (12.5 %)	0 (0 %)	2 (18.2 %)	1 (25 %)	
**Parity**						0.803
- Para = 0 (n, %)	44 (86.3 %)	28 (87.5 %)	4 (100 %)	8 (72.73 %)	4 (100 %)	
- Para = 1 (n, %)	4 (7.8 %)	2 (6.3 %)	0 (0 %)	2 (18.2 %)	0 (0 %)	
- Para ≥ 2 (n, %)	3 (5.9 %)	2 (6.2 %)	0 (0 %)	1 (9.1 %)	0 (0 %)	
**Abortions**						0.830
- Abortions = 0 (n, %)	44 (86.3 %)	27 (84.4 %)	4 (100 %)	9 (81.8 %)	4 (100 %)	
- Abortions = 1 (n, %)	4 (7.8 %)	2 (6.3 %)	0 (0 %)	2 (18.2 %)	0 (0 %)	
- Abortions ≥ 2 (n, %)	3 (5.9 %)	3 (9.4 %)	0 (0 %)	0 (0 %)	0 (0 %)	
**Family History of Malformations**						1
- No (n, %)	45 (90 %)	27 (87.1 %)	4 (100 %)	10 (90.9 %)	4 (100 %)	
- Yes (n, %)	5 (10 %)	4 (12.9 %)	0 (0 %)	1 (9.1 %)	0 (0 %)	
**Correction of Malformation**						0.297
- No (n, %)	31 (77.5 %)	19 (82.6 %)	1 (50 %)	7 (63.6 %)	4 (100 %)	
- Yes (n, %)	9 (22.5 %)	4 (17.4 %)	1 (50 %)	4 (36.4 %)	0 (0 %)	
**Symptomatic**						0.382
- No (n, %)	15 (29.4 %)	11 (34.4 %)	0 (0 %)	4 (36.4 %)	0 (0 %)	
- Yes (n, %)	36 (70.6 %)	21 (65.6 %)	4 (100 %)	7 (63.6 %)	4 (100 %)	
**Malformation as Part of Syndrome**						< 0.001
- No (n, %)	43 (84.3 %)	31 (96.9 %)	0 (0 %)	9 (81.8 %)	3 (75 %)	
- Yes (n, %)	8 (15.7 %)	1 (3.1 %)	4 (100 %)	2 (18.2 %)	1 (25 %)	

Median and IQR are provided for metric variables (with more than 3 observations per group). Absolute and relative frequencies are computed for nominal and ordinal variables in the total study population as well as per study center. Categories larger than or equal to 2 are merged for para, gravidity, and number of abortions. Note that only observations with valid values are used to calculate percentage values. P-values are reported for Kruskal-Wallis-Test on location differences of age, time to diagnosis and patients investigated between study centers and for Fisher’s exact test on association between study center and categorical variables. Symptomatic patients experienced amenorrhea, infertility, pain or miscarriages.

**Fig. 1:**
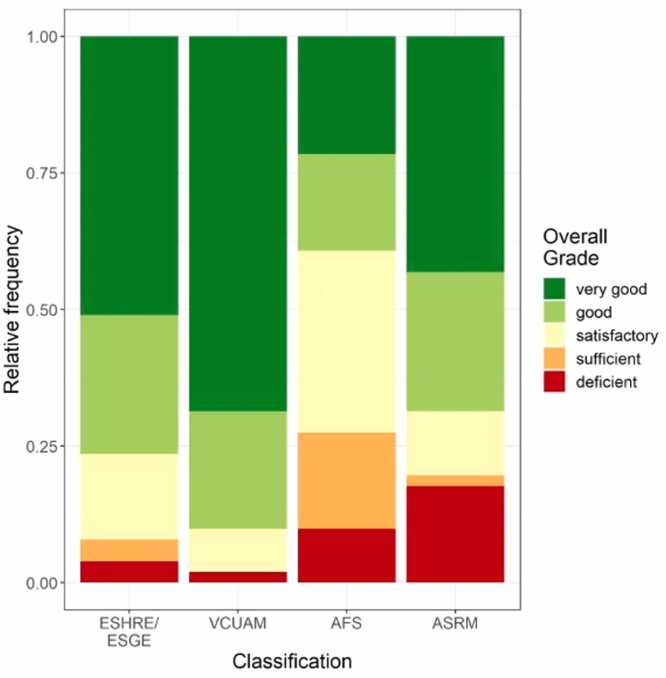
Overall grading for usability of classifications. Relative frequencies of the overall grading of the classification methods: ESHRE/ESGE, VCUAM, AFS and ASRM MAC2021.

Fifty-one patients with female genital malformations were classified with the AFS and reclassified using the ASRM classification. Usability is evaluated for each classification system in total and separately for each compartment. Fig. 1 summarizes clinician’s grading for overall usability of each classification system (ESHRE/ESGE, VCUAM, AFS and ASRM). The organ-based classifications VCUAM and ESHRE/ESGE were rated highest, with overall grades of “very good” or “good” in 90.2 % of cases (n = 46/51) for the VCUAM classification and 76.5 % (n = 39/51) for the ESHRE/ESGE classification. The VCUAM and ESHRE/ESGE classifications were rated “deficient” in 2 % (n = 1/51) and 3.9 % (n = 2/51) of cases, respectively. The AFS classification was rated “very good”/“good” in 39.2 % of cases (n = 20/51) and the ASRM classification was rated “very good”/“good” in 68.6 % (n = 35/51). The rating “deficient” was given in 9.8 % of cases (n = 5/51) for the AFS and in 17.7 % (n = 9/51) for the ASRM classification.

Subjective evaluation of reproducibility was evaluated with the question “Do you think that another person could accurately reproduce the diagnosed deformity using the classification item created?” The AFS classification was assessed as reproducible in 64.7 % of cases (n = 33/51). The ASRM was rated as reproducible in 78.4 % (n = 40/51). The ESHRE/ESGE classification system was rated as reproducible in 86.3 % of cases (n = 44/51) and the VCUAM classification in 96.1 % (n = 49/51).

**Fig. 2:**
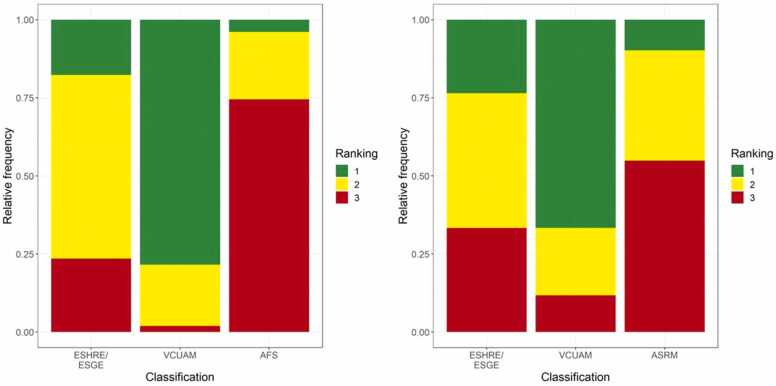
Ranking of classifications. Relative frequencies of the old ranking of classification methods (left) and relative frequencies of the new ranking of classification methods (right).

The investigators were asked to rank the classifications in relation to clinical practicality, with “1” representing the most practical and “3” the least practical. An overview of the individual rankings using the AFS versus the ASRM classification is provided in Fig. 2. The AFS classification was ranked “1” in 3.9 % of cases (n = 2/51). The ESHRE/ESGE was ranked “1” in 17.7 % (n = 9/51) and VCUAM was ranked “1” in 78.4 % (n = 40/51). After reclassification of the AFS into the ASRM classification, ASRM was ranked “1” in 9.8 % (n = 5/51), ESHRE/ESGE in 23.5 % (n = 12/51), and VCUAM in 66.7 % of cases (n = 34/51). The AFS was ranked “3” in 74.5 % (n = 38/51), ESHRE/ESGE in 23.5 % (n = 12/51), and VCUAM in 2 % of cases (n = 1/51). After reclassification of the AFS into the ASRM classification, ASRM was ranked “3” in 54.9 % (n = 28/51), ESHRE/ESGE in 33.3 % (n = 17/51), and VCUAM in 11.8 % of cases (n = 6/51).

**Fig. 3:**
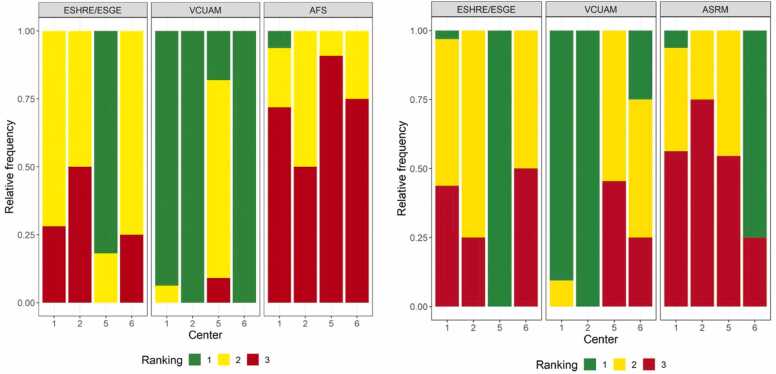
Ranking by study center. Relative frequencies of rankings by study center per classification method. The left side represents the old rankings, including the AFS classification; the right side represents the new rankings after reclassification for the ASRM MAC2021.

**Fig. 4:**
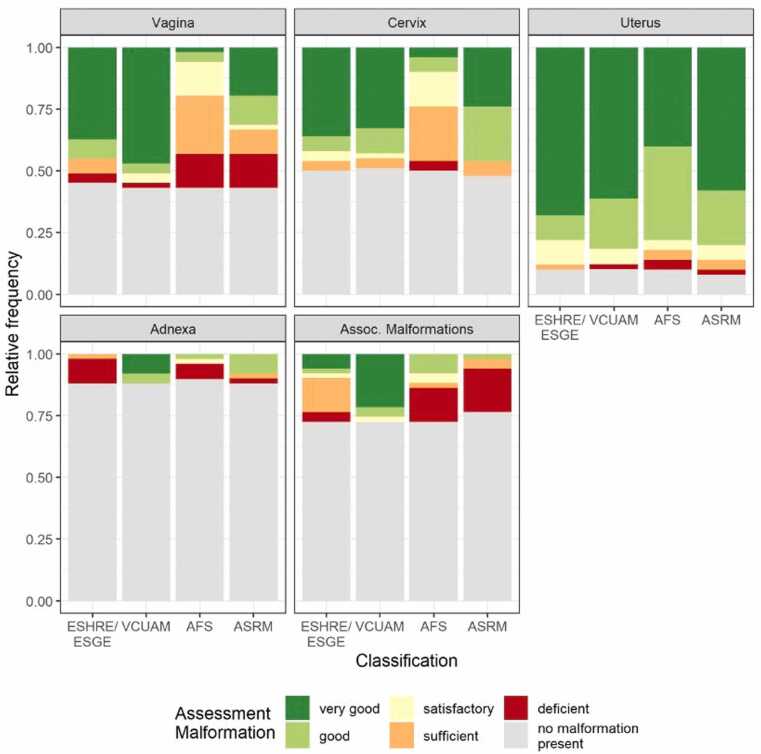
Assessment of compartments. Relative frequencies of assessments of malformations in the vagina, cervix, uterus, adnexa, and associated malformations by classification method.

The additional stacked bar charts in Fig. 3 show the relative frequencies of rankings ("one" to “three”) for the overall classifications stratified by study center. Additionally, each compartment of female genital malformations was individually rated for usability and ranked for clinical practicality by the investigators. Fig. 4 shows the relative frequencies of assessment of usability (“very good” to “deficient”) in each of the compartments with the four different classification systems. The classification systems were not rated for a specific compartment in cases with no malformation present.

### Vagina

For vaginal malformations in terms of usability, the AFS classification was rated “very good”/“good” in 10.3 % (n = 3/29) of the patients. The ASRM classification was rated “very good”/“good” in 55.2 % (n = 16/29). The ESHRE/ESGE classification was rated “very good”/“good” in 82.1 % (n = 23/28) and the VCUAM classification in 89.7 % (n = 26/29) of cases. A rating of “deficient” was given to both the AFS and the ASRM classifications in 24.1 % of cases (n = 7/29) for assessing vaginal malformations, with 7.1 % (n = 2/28) for the ESHRE/ESGE and 3.5 % (n = 1/29) for the VCUAM classification.

### Cervix

For assessing cervical malformations, a usability rating of “very good”/“good” was given to the AFS in 20 % (n = 5/25) of cases and to the ASRM in 88.5 % (n = 23/26). The ESHRE/ESGE classification was rated “very good”/“good” in 84 % (n = 21/25) and the VCUAM classification in 87.5 % (n = 21/24). The grade “deficient” in the assessment of cervical malformation was given to the AFS classification in 8 % (n = 2/25) and to the ASRM classification in 0 % (n = 0/26). The ESHRE/ESGE and VCUAM classifications were rated “deficient” in 0 % (n = 0/25 and n = 0/24).

### Uterus

When rating the usability of the different classification system for the assessment of uterine malformations, a rating of “very good”/“good” was given to the AFS in 86.7 % (n = 39/45) and to the ASRM in 87 % (n = 40/46). The ESHRE/ESGE classification was rated “very good”/“good” in 86.7 % (n = 39/45) and the VCUAM classification in 91 % (n = 40/44). A rating of “deficient” was given to the AFS in 4.4 % (n = 2/45) and to the ASRM in 2.2 % (n = 1/46). ESHRE/ESGE was rated “deficient” in 0 % (n = 0/45) and VCUAM was rated “deficient” in 2.3 % (n = 1/44).

### Adnexa

When assessing adnexal malformations, a rating of “very good”/“good” was given to the AFS classification in 20 % (n = 1/5) and to the ASRM classification in 66.7 % (n = 4/6). A rating of “very good”/“good” was given to the ESHRE/ESGE classification in 0 % (n = 0/6). The VCUAM classification was rated “very good”/“good” in 100 % (n = 6/6). A “deficient” rating using the AFS was given in 60 % of cases (n = 3/5). After reclassification to the ASRM, it was rated “deficient” in 16.7 % (n = 1/6). The ESHRE/ESGE system was rated “deficient” in 83.3 % (n = 5/6) and VCUAM was graded “deficient” in 0 % (n = 0/6).

### Associated malformations

The VCUAM classification was considered the most practical system for assessing associated malformations, whereas the ASRM and AFS classifications were perceived as the least practical. The VCUAM system received a rating of "very good" or "good" in 92.9 % of cases (n = 13/14), with no instances rated as "deficient" (0 %, n = 0/14). In comparison, both the AFS and ESHRE/ESGE classifications were rated "very good"/"good" in 28.6 % of cases (n = 4/14), while the ASRM classification achieved this rating in only 8.3 % of cases (n = 1/12). With regard to low practicality, the ASRM classification was rated as "deficient" in 75 % of cases (n = 9/12), the AFS in 50 % (n = 7/14), and the ESHRE/ESGE in 14.3 % (n = 2/14).

## Discussion

In the present study, the ASRM Müllerian Anomalies Classification 2021 was better accepted by clinicians in their everyday practice in comparison with the American Fertility Society classification. As discussed by Ludwin et al., the ASRM MAC2021 task force introduced meaningful improvements to classification clarity and anatomical comprehensiveness [20]. Overall, the ASRM system was rated “very good” by twice as many respondents and was considered more clinically practical, reproducible and useful for routine assessment across most compartments.

ASRM MAC2021 was ranked as the most clinically practical classification more than twice as often as AFS with higher subjectively rated reproducibility. The ASRM classification showed substantial improvements in the evaluation of vaginal, cervical, and adnexal anomalies. For vaginal anomalies, ASRM received a “very good”/“good” rating in more than half of cases, a significant improvement compared to AFS. This is mostly attributed to ASRM’s inclusion of isolated vaginal anomalies such as transverse and longitudinal septa and vaginal agenesis, which were previously unaddressed in AFS. Unfortunately, some female genital malformations, such as urogenital sinus or anomalies of the hymen, are not included in either in the AFS or the ASRM classification systems. The ASRM classification outperformed AFS for cervical anomalies and adnexal anomalies, reflecting the more comprehensive structural categorization and updated terminology in ASRM MAC2021. The ASRM classification was rated “very good”/“good” more often than the AFS classification in the diagnosis of adnexal malformations and the percentage of “deficient” ratings declines considerably after cases are reclassified from the AFS to ASRM systems. Of note, the number of patients with adnexal malformation was small and should therefore cautiously be interpreted.

Both the ASRM and AFS systems perform well in the classification of uterine malformations. With almost ninety percent of the respondents rating them “very good” or “good,” these proportions are similar to those for ESHRE/ESGE and VCUAM, whereas “deficient” ratings are less frequent.

However, while ASRM MAC2021 presents notable advancements, our results also demonstrate that it is surpassed by the VCUAM and ESHRE/ESGE classification systems, especially in the evaluation of complex and associated anomalies. The VCUAM classification was consistently rated highest across nearly all compartments, with over ninety-five per cent of respondents finding it reproducible and the majority ranking it as the most clinically practical. VCUAM’s structural advantages likely explain its superior performance. Unlike AFS and ASRM, VCUAM uses a compartment-based approach similar to the TNM system used in oncology, separately classifying anomalies of the vagina, cervix, uterus, adnexa and associated malformations, such as malformations of the renal system. This modular structure allows clinicians to more precisely describe combinations of malformations, including rare or complex cases. Importantly, VCUAM explicitly includes associated malformations — a key feature lacking in ASRM MAC2021. This likely explains why ASRM performed poorly in this domain (only 8.3 % rated “very good”/“good”), while VCUAM received favorable ratings in over ninety per cent of cases. The AFS classification, though outdated, still outperformed ASRM for associated anomalies, possibly due to its brief mention of “additional findings.” Pfeifer et al. aimed to include all categories of malformations [9]. However, associated malformations are not included in the proposed diagnostic tool.

In our study cohort eleven patients underwent MRI in addition to ultrasound. Examination using MRI was indicated in patients when transvaginal gynecological ultrasound was not possible, such as young virgo patients. Other indications for MRI were cases with associated anomalies, mainly of the renal system, and in very few cases of complex anomalies, such as sinus urogenitalis. Transvaginal gynecologic ultrasound was directly performed by the study investigators themselves, who were all trained in the diagnosis and treatment of patients with müllerian anomalies and who did additionally perform gynecological examination. Therefore, the study team concluded that generally the combination of gynecologic examination, transvaginal and transabdominal ultrasound is sufficient for diagnosing female genital malformations. In contrast, some countries rely on ultrasound examinations performed by ultrasound technicians. In situations like these, relevant information obtained via gynecological examination could be missing for ascertaining the correct diagnosis. All MRI results were reviewed by the respective investigators of the study team, but the study refrained from an in-depth analysis of MRI data, acknowledging that MRI examination is only relevant for diagnosis of müllerian anomalies in a very small percentage of cases.

Differences in classification systems can significantly impact clinical decision-making, interdisciplinary communication and patient outcomes. Incomplete or ambiguous classification — such as failure to document cervical or associated anomalies — can result in missed diagnoses or inappropriate management. For instance, accurate identification of a cervical or uterine anomaly is crucial when planning fertility-preserving surgery or assisted reproductive techniques. Similarly, associated renal anomalies may require interdisciplinary follow-up with nephrology or urology, and their omission from the classification system can lead to insufficient patient care.

The ASRM Müllerian Anomalies Classification clearly improved the diagnosis of women with genital malformations and in terms of reproducibility and usability was rated as superior to the American Fertility Society (AFS) classification. Other authors have already called for a consensus on classification of müllerian anomalies [18] and the need for continuous improvement of classification systems has already been emphasized when the American Fertility Society classification was first published [1]. Furthermore, consistent and detailed classification supports effective communication across specialties (e.g., radiology, surgery, reproductive medicine), enhances documentation for patient records and facilitates planning of multidisciplinary case conferences. On a broader scale, unifying classification systems is vital for epidemiological research, registries and clinical trials, enabling accurate case aggregation and outcome comparisons. Given the current fragmentation among classification systems, there is an urgent need for integration and global consensus. A unified system should combine the compartment-based structure of VCUAM with the international familiarity and anatomical clarity of ASRM and include associated malformations as a standard component. The ideal structure should facilitate input from multiple specialties (e.g., nephrology, radiology) and remain flexible enough to classify atypical and mixed anomalies while avoiding excessive complexity that limits routine use.

The path toward a global consensus likely requires collaboration between major societies such as ASRM, ESHRE/ESGE and others alongside contributions from centers with high volumes of patients with müllerian anomalies. Cooperation between study groups working on diagnosis and therapy of müllerian anomalies should aim at a unified and complete classification system to enable international collaboration and ongoing improvement of patient care. A Delphi consensus process, building on multicenter input and real-world usability testing (such as our study), could be a pragmatic approach. Additionally, establishing an international registry using a unified classification system could promote iterative refinement and validation.

### Limitations

This study is the only published comparison of the AFS and ASRM classifications for female genital malformations. Its multicenter, international design allowed for inclusion of diverse cases examined by experienced clinicians. Nonetheless, some limitations should be acknowledged. First, the practicality and reproducibility ratings were subjective and based on the individual experience of each clinician. The number of patients evaluated per investigator varied, and the study design did not account for interobserver variability. Additionally, the sample size for some compartments (e.g., adnexa, associated anomalies) was small, limiting generalizability in those areas.

We deliberately chose a subjective approach, reflecting real-world applicability and daily diagnostic scenarios. While interrater reliability metrics are valuable, they require standardized cases and controlled settings, which were outside the scope of this pragmatic evaluation. Future studies should aim to quantify interobserver agreement using predefined cases and explore how different classification systems affect clinical decision-making in prospective patient cohorts.

## CRediT authorship contribution statement

**Julia Lastinger:** Writing – original draft, Project administration, Conceptualization. **Natalia Palasz:** Writing – original draft, Investigation, Data curation. **Peter Oppelt:** Writing – review & editing, Supervision, Conceptualization. **Galymzhan Toktarbekov:** Investigation, Data curation. **Milan Terzic:** Writing – review & editing, Data curation, Conceptualization. **Raimund Stein:** Writing – review & editing, Data curation. **Katharina Rall:** Writing – review & editing, Data curation. **Helga Wagner:** Formal analysis. **Philipp Hermann:** Formal analysis. **Stephanie Kiblboeck:** Writing – original draft, Formal analysis, Data curation, Conceptualization.

## Declaration of Competing Interest

The authors declare that they have no known competing financial interests or personal relationships that could have appeared to influence the work reported in this paper.
